# Estimating excess septicaemia mortality and hospitalisation burden associated with influenza in Hong Kong, 1998 to 2019

**DOI:** 10.1017/S0950268822000760

**Published:** 2022-04-27

**Authors:** Jessica Y. Wong, Chung-Mei M. Cheung, Helen S. Bond, Justin K. Cheung, Huizhi Gao, Vicky J. Fang, Eric H. Y. Lau, Benjamin J. Cowling, Peng Wu

**Affiliations:** 1 WHO Collaborating Centre for Infectious Disease Epidemiology and Control, School of Public Health, Li Ka Shing Faculty of Medicine, The University of Hong Kong, Hong Kong Special Administrative Region, China; 2 Basildon University Hospital, Basildon, Essex, UK; 3 Laboratory of Data Discovery for Health Limited, Hong Kong Science Park, New Territories, Hong Kong Special Administrative Region, China

**Keywords:** Influenza, death, hospitalisation

## Abstract

Influenza virus infections can lead to a number of secondary complications, including sepsis. We applied linear regression models to mortality and hospital admission data coded for septicaemia from 1998 to 2019 in Hong Kong, and estimated that septicaemia was associated with an annual average excess mortality rate of 0.23 (95% CI 0.04–0.40) per 100 000 persons per year and an excess septicaemia hospitalisation rate of 1.73 (95% CI 0.94–2.50) per 100 000 persons per year. The highest excess morbidity and mortality was found in older adults and young children, and during influenza A(H3N2) epidemics.

## Introduction

The majority of patients with seasonal influenza present with mild upper respiratory symptoms and have a self-limiting disease, but some develop more severe diseases requiring hospitalisation. Among patients hospitalised with influenza, one particularly severe complication over the clinical course is sepsis, defined as systemic organ dysfunction as a result of a dysregulated host response to infection [1].

It is well recognised that the morbidity and mortality burden of influenza is greater in young children, older people and those with pre-existing co-morbidities. There is a clear link between influenza virus infection and resultant sepsis [2]. Sepsis makes up to 73% of all hospitalisation admissions related to critical illness caused by seasonal and pandemic influenza [3] and leads to a substantial mortality burden. Currently, there are limited data on the contribution of sepsis to the overall health impact of influenza. Severe influenza is often underdiagnosed [4], and influenza infection is not always specifically coded on death certificates or hospital records outside of pandemic periods [5]. Here, we modelled time series of hospitalisations and deaths in the Hong Kong population from 1998 through 2019 to estimate the excess hospitalisation and mortality rates from influenza-associated septicaemia stratified by age group and influenza virus type/subtype.

## Methods

### Sources of data

Weekly deaths from 1998 to 2019 were obtained from the Census and Statistics Department of the Hong Kong. Weekly hospital admissions were collected from the Hospital Authority for patients admitted to all local public hospitals, which cover approximately 90% of hospital bed days in Hong Kong. Septicaemia deaths and hospital admissions were coded according to the International Classification of Diseases, Ninth Revision (ICD-9: 038) and Tenth Revision (ICD-10: A40–A41). The mid-year population for each year from 1998 through 2019 were obtained from the Census and Statistics Department and used as the denominators for the estimation of mortality and hospitalisation rates.

Weekly surveillance data of outpatient consultations due to influenza-like illness (ILI), defined as fever with cough and/or sore throat, were reported by the Hong Kong Centre for Health Protection. Weekly laboratory data on influenza virus detections by type/subtype were reported by the Public Health Laboratory Services. We multiplied the weekly ILI data with the weekly proportion of laboratory specimens tested positive for each type/subtype of influenza, and used them as proxies measures for influenza virus activity in the community (denoted as ILI+) [6, 7]. We constructed ILI+ proxies for influenza A(H1N1), influenza A(H3N2), and influenza B. We used the weekly hospitalisation rate of acute bronchiolitis associated with the respiratory syncytial virus (RSV) in children <1 year of age as the proxy measure for RSV activity. Meteorological data including daily temperature and relative humidity were obtained from the Hong Kong Observatory.

### Statistical analysis

We applied linear regression models to investigate the underlying association between weekly septicaemia hospitalisation or mortality rates and influenza activity in the community, as represented by the ILI+ proxies. We used a generalised additive model (GAM) to reflect an additive relation between influenza activity and mortality or hospitalisation rates, which might be more plausible than models assuming multiplicative increases between certain outcomes or complications and influenza activity [7]. Such a model also adopts the flexibility for the baseline rates of mortality and hospitalisations not associated with influenza, which was suitable for year-round circulation of influenza activity in subtropical regions [8, 9]. We included temperature, absolute humidity and RSV activity (described above) as covariates in the regression models. We excluded February-September 2003, which was affected by the Severe Acute Respiratory Syndrome epidemic. We included a covariate in the regression models to account for a change in sentinel surveillance practice in Hong Kong during and after the 2009 influenza A(H1N1)pdm09 pandemic. We also included a covariate into the models for mortality rates to account for the impact of the transition of a coding system (i.e. from ICD-9 to ICD-10) for mortality data in Hong Kong since 2001 [7]. Another covariate was included into the models for hospitalisation rates to account for the impact of public holidays on hospital admissions [6].

Given the likely delay between onset of influenza illness and death, we specified a lag of 1 week between influenza activity and mortality rates, and a lag of 0 weeks between influenza activity and hospitalisation rates. In sensitivity analyses, we lagged influenza activity by 0, 2 and 3 weeks for mortality and 1 and 2 weeks for hospitalisation. The 95% confidence intervals (CIs) for excess hospitalisation or mortality rates were estimated with a bootstrap approach. The influenza-associated excess hospitalisations and death rates from septicaemia were estimated by subtracting the predicted hospitalisation and death rates under the model with ILI+ proxies set to zero from the predicted rates with ILI+ proxies set to their observed values [7]. All statistical analyses were conducted in R version 4.0.3 (R Foundation for Statistical Computing, Vienna, Austria).

## Results

Between 1 January 1998 and 28 December 2019, we studied a total of 16 014 septicaemia deaths and 179 116 septicaemia hospitalisations, with the population of Hong Kong increasing from 6.52 million persons in 1998 to 7.43 million in 2019. Of the 22 years covered, most of the years had prolonged periods of influenza virus circulation, with more than one distinct epidemic wave in the years 2000, 2003, 2008 and 2016, and prolonged influenza activities for more than 5 months in some years including 2002 and 2004 (Fig. S2). Temperature and absolute humidity exhibit well-defined seasonal trends throughout the study period. While weekly mean temperature ranges between 10.3 °C and 30.7 °C, weekly mean absolute humidity ranges between 4.2 g/m^3^ and 24.7 g/m^3^ (Fig. S3).

Using regression analysis, we estimated that influenza was associated with an annual excess septicaemia mortality rate of 0.23 (95% CI 0.04–0.40) per 100 000 persons per year, and an excess septicaemia hospitalisation rate of 1.73 (95% CI 0.94–2.50) per 100 000 persons per year (Table 1). This corresponded to an average of 16 (95% CI 3–28) excess septicaemia deaths per year and an average of 120 (95% CI 65–174) excess septicaemia hospitalisations per year, which were 2.2% (95% CI 0.4%–3.8%) of all septicaemia deaths, and 1.5% (95% CI 0.8%–2.1%) of all septicaemia hospitalisations, reported in Hong Kong during the study period.

**Table 1.** Average type and subtype-specific annual excess septicaemia mortality and hospitalisation rates in Hong Kong, 1998 to 2019

	Average excess mortality and hospitalisation rate (per 100 000 population per year)			
	A(H1N1)	A(H3N2)	B	All influenza
Mortality	0.04 (−0.07 to 0.14)	0.02 (−0.10 to 0.15)	0.18 (0.03–0.31)	0.23 (0.04–0.40)
Hospitalisation	−0.13 (−0.55 to 0.31)	1.19 (0.65–1.69)	0.68 (0.16–1.24)	1.73 (0.94–2.50)

Amongst the subtypes of influenza studied, influenza B and influenza A(H3N2) were associated with the greatest average septicaemia mortality and hospitalisation burden, with an excess mortality rate of 0.18 (95% CI 0.03–0.31) per 100 000 persons per year and an excess hospitalisation rate of 1.19 (95% CI 0.65–1.69) per 100 000 persons per year (Table 1). In addition, influenza B was also associated with a relatively higher excess hospitalisation rate of 0.68 (95% CI 0.16–1.24) per 100 000 persons per year (Table 1).

The highest excess septicaemia mortality was found in the age group ≥65 y, with a rate of 1.53 (95% CI 0.10–2.86) per 100 000 persons per year (Table S2). This burden was higher amongst females (2.53, 95% CI 0.79–4.21) than males (0.27, 95% CI −1.50 to 2.01). The influenza-associated annual average excess septicaemia hospitalisation rates were estimated to be 11.39 (95% CI 3.87–18.10) per 100 000 persons per year and 7.09 (95% CI 2.85–12.92) per 100 000 persons per year in individuals at age of 0–4 y and ≥65 y, respectively (Table S3). The excess hospitalisation rate in females aged ≥65 y (7.55, 95% CI 2.14–14.47 per 100 000 persons per year) was slightly higher than males in the same age group (6.79, 95% CI 0.91–13.46 per 100 000 persons per year). In the sensitivity analyses, point estimates of the excess mortality and hospitalisation rates were similar when alternative choices of lag were assumed between influenza activity and the studied outcomes (Tables S4–S5).

## Discussion

Our study applied statistical models to estimate excess hospitalisation and mortality rates resulting from influenza-associated septicaemia. We used a linear regression model to estimate disease burden, which accounts for the climate in Hong Kong where there are multiple waves of influenza activity annually, and can account for covariates such as RSV activity, temperature and humidity [7]. We estimated that influenza is associated with 0.23 (95% CI 0.04–0.40) excess septicaemia deaths and 1.73 (95% CI 0.94–2.50) hospitalisations per 100 000 persons per year. This equates to on average 16 (95% CI 3–28) excess deaths and 120 (95% CI 65–174) excess hospitalisations resulting from influenza-associated septicaemia every year. However, the admission data indicated that there were on average only 15 (range: 1, 62) inpatients hospitalised with septicaemia as the primary diagnosis had a laboratory-confirmed influenza infection each year in Hong Kong over the same time period.

Wu *et al*. [6] found that influenza was associated with 6.27 excess respiratory deaths and 184 excess hospitalisations per 100 000 persons per year from January 1998 through June 2013 in Hong Kong. Our analysis showed that the influenza-associated excess mortality and morbidity from septicaemia were about 3.7% and 0.9% of the influenza-associated excess respiratory mortality and morbidity, respectively. While influenza is well recognised to have a high respiratory disease burden [6, 7], it is also possible that the burden of septicaemia has been underestimated, as sequelae of severe sepsis, such as acute respiratory distress syndrome [10] and circulatory failure might not have been coded under septicaemia.

The existing literature has limited age and subtype-specific data on influenza-associated septicaemia deaths and hospitalisations. Despite differences in population demographics and seasonal baselines, the average estimates of excess sepsis hospitalisation rate from seasonal influenza in the United States were similar to those presented here [11]. Another study from New York City also reported a significant contribution to the hospitalisation burden in older adults, ranging from 8.8 to 38.7 per 100 000 in individuals aged 65–74 y and ≥ 75 y respectively [8]. We find that the burden of influenza-associated septicaemia death is highest in the ≥65 y age group, and much greater in females than males. This is consistent with the current evidence base demonstrating that the risk of serious illness and death is higher in the older age group [6]. The identified excess disease burden from influenza virus infection could be due to the deterioration and decompensation of existing co-morbidities in older individuals after infection with therefore a higher risk of severe complications.

Influenza-associated excess hospitalisation rates followed a *U*-shaped pattern, with the highest rates at the extremes of age (<1 y and ≥65 y). This is observed in the current analysis on septicaemia as well as previous studies on influenza-associated respiratory illnesses [7]. It is possible that, despite having a less robust immunity system compared to other age groups, infants and young children might have better physiological reserves with few co-morbidities, and younger patients are also more likely to be seen and treated promptly, particularly for infants who are also more likely to be admitted for observation as part of clinical risk management [12].

Influenza A (H3N2) is associated with the highest excess septicaemia hospitalisation rates across age and sex groups from our study. The higher disease burden associated with A(H3N2) could be due to greater virulence of the virus, a faster mode of spread, a higher infection rate, a more rapid antigenic shift and potential interference with other co-circulating virus strains, complicating the disease course of infections with other subtypes [7, 13, 14]. However, we found that in relation to excess septicaemia mortality, Influenza B is responsible for the majority of deaths in individuals ≥65 y. This was not reported with the excess respiratory deaths from influenza infection. Further studies might be needed to explore the link between causes of death and influenza virus types and subtypes.

There are some limitations to our study. First, our methodology relied on community surveillance data of influenza activity. We used a proxy which is the product of recorded cases of ILI and laboratory tested influenza samples, taking into account the information on the detection of influenza viruses as well as the variations in ILI activities in the community [7]. In addition, our model included RSV as a covariate as it has been shown to interact with the influenza virus where there had been concurrent waves of influenza and RSV and RSV may also lead to substantial hospitalisation and mortality in a population [15]. Solid conclusions based on our estimates have been challenging due to wide confidence intervals resulting from the small number of events that occurred in a relatively small population, along with some negative estimates. However, results presented in the Appendix have displayed evidence of statistical significance by subtype when CIs are non-overlapping. While some estimates are negative, they do not significantly differ from zero. Future efforts at statistical modelling of influenza disease burden may include other potential confounders such as other viral activity and healthcare usage, etc., and evaluate their impact on the modelling of influenza-associated disease burden.

In conclusion, our study identified excess deaths and hospitalisations from septicaemia associated with influenza infections, and population groups at a higher risk of the disease burden in a location with prolonged periods of influenza activity each year.

## Data Availability

The data that support the findings of this study are available from the Census & Statistics Department and the Hong Kong Hospital Authority. Restrictions apply to the availability of these data, which were used under license for the current study, and so are not publicly available. Data are however available via request from the Census & Statistics Department or the Hong Kong Hospital Authority.

## Supplementary material

For supplementary material accompanying this paper visit http://doi.org/10.1017/S0950268822000760. click here to view supplementary material
