# Supplementary material for: Estimating excess septicaemia mortality and hospitalisation burden associated with influenza in Hong Kong, 1998 to 2019
Source: Epidemiol Infect. 2022 Apr 27;150:e101. doi: 10.1017/S0950268822000760 (PMC9128349; doi:10.1017/S0950268822000760)
Supplement: Supplementary file 1 [file S0950268822000760sup001.docx]

# APPENDIX

# Estimating excess septicemia mortality and hospitalization burden associated with influenza in Hong Kong, 1998 to 2019

This appendix provides additional information on the statistical methods used in this study.

**1. Generalized additive model**

We used generalized additive models for estimating influenza-associated excess mortality and hospitalization for septicemia causes. The following two equations show the models used for weekly death and hospitalization rates:

*μ_D_ = β_1_*s(Week Index, k=43) + β_2_* (h1n1*ILI) + β_3_* (h3n2*ILI) + β_4_* (B*ILI) + β_5_* (ph1n1*ILI) + β_6_* (ph1n1*ILI*Pan) + β_7_* (RSV*ILI) + β_8_*Tem + β_9_* Tem ^2^ + β_10_* Tem ^3^ +
β_11_* AH + β_12_*AH^2^ + β_13_*AH^3^ + β_14_*Cod*

*Death rate ~ Normal(μ_D_, τ_D_^2^)*

*μ_H_ = α_1_*s(Week Index, k=43) + α_2_* (h1n1*ILI) + α_3_* (h3n2*ILI) + α_4_* (B*ILI) + α_5_* (ph1n1*ILI) + α_6_* (ph1n1*ILI*Pan) + α_7_* (RSV*ILI) + α_8_* Tem + α_9_* Tem ^2^ + α_10_* Tem ^3^ + α_11_*AH + α_12_*AH^2^ + α_13_*AH^3^ + α_14_*Hol*

*Hospitalization rate ~ Normal(μ_H_, τ_H_^2^)*

where *s(Week Index, k=43)* is a spline function on calendar weeks of the study period to account for seasonal patterns of population death rate and hospitalization rate. *Pan* is a dummy variable for potential changes in healthcare practices and health seeking behavior in response to the first wave of the 2009 pandemic as used in our previous studies. *Tem* and *AH* refer to the mean weekly temperature and absolute humidity, respectively, given that ambient temperature and humidity have been indicated to be associated with virus survival and transmissibility. *Cod*, a dummy variable, accounts for the change in disease coding system for mortality data from ICD-9 (1998-2000) to ICD-10 (2001 and thereafter) in Hong Kong. *Hol* refers to the dummy variable accounting for possible differences in health seeking behavior of the public during public holidays.
*β_1_ - β_14_* and *α_1_ - α_14_* are the regression coefficients for variables included in the mortality model and the hospitalization model, respectively.

**2. Model fit and choice of lags**

Model fit was assessed using residuals from fitted models. No apparent pattern is identified in the residuals and the estimated autocorrelation function (ACF) of the residuals for the two statistical models (mortality and hospitalization) for all ages and all flu subtypes (Figure S1), and results suggested that the models under the optimal choices of lag fitted well to the observed data (Figure S2). In addition to ACF, we also assessed model fit using the mean squared error values (MSE) from the different models. The comparison of MSE for all possible mortality and hospitalization models is shown in Table S1, and results demonstrated that the mortality model with a 1-week lag and hospitalization model with a 0-week lag provided the best fit due to their respectively lowest MSE values.

As part of our analysis, we fitted the mortality and hospitalization model with different choice of lags, ranging from 0 to 3 weeks. Our main analysis focused on obtaining estimates from the models with the optimal choices of 0-week lag for hospitalization and 1-week lag for mortality (Tables S2-S3), while alternative lags were assessed in our sensitivity analyses (Tables S4-S5). The hospitalization model with a 3-week lag was not considered in the supplementary analysis since influenza patients are symptomatic and that they are usually hospitalized within a short period of time.

Figure S1. Autocorrelation function plot based on residuals across models with different choice of lags.

Table S1. MSE for mortality and hospitalization models with different choice of lags.

| **Lag** | **Mortality model** | **Hospitalization model** |
| --- | --- | --- |
| 0-week | 0.00304578 | **0.04558948** |
| 1-week | **0.003042413** | 0.04602886 |
| 2-week | 0.003071996 | 0.04604204 |
| 3-week | 0.003079536 | NA |

NA=not applicable

Figure S2. (A) Weekly septicemia mortality rates per 100,000 persons in Hong Kong: observed, fitted and baseline, 1998 to 2019. (B) Weekly septicemia hospitalization rates per 100,000 persons in Hong Kong: observed, fitted and baseline, 1998 to 2019. (C) Weekly RSV activity (grey line) and influenza activity by subtype: influenza A(H1N1) (red line), A(H3N2) (green line), and influenza B (blue line).

Table S2. Average type and subtype-specific annual excess septicemia mortality rates in different sex and age groups in Hong Kong, 1998 to 2019, assuming 1-week lag between influenza incidence and mortality.

|  | Average excess mortality rate (per 100,000 population per year) | | | | | | | |
| --- | --- | --- | --- | --- | --- | --- | --- | --- |
| Virus | 0-4 y | (95% CI) | 5-64 y | (95% CI) | ≥65 y | (95% CI) | All Ages | (95% CI) |
| **Male** |  |  |  |  |  |  |  |  |
| A(H1N1) | -0.14 | (-0.39, 0.17) | -0.02 | (-0.09, 0.05) | -0.44 | (-1.34, 0.45) | -0.04 | (-0.18, 0.08) |
| A(H3N2) | 0.09 | (-0.27, 0.51) | 0.02 | (-0.05, 0.11) | 0.46 | (-0.57, 1.63) | 0.02 | (-0.12, 0.21) |
| B | -0.17 | (-0.57, 0.25) | 0.08 | (-0.01, 0.17) | 0.25 | (-1.09, 1.50) | 0.08 | (-0.08, 0.27) |
| All influenza | -0.22 | (-0.84, 0.39) | 0.08 | (-0.05, 0.22) | 0.27 | (-1.50, 2.01) | 0.06 | (-0.16, 0.32) |
|  |  |  |  |  |  |  |  |  |
| **Female** |  |  |  |  |  |  |  |  |
| A(H1N1) | 0.06 | (-0.16, 0.37) | 0.03 | (-0.01, 0.06) | 0.45 | (-0.44, 1.36) | 0.09 | (-0.03, 0.21) |
| A(H3N2) | -0.18 | (-0.48, 0.21) | 0.01 | (-0.04, 0.05) | 0.42 | (-0.66, 1.52) | 0.07 | (-0.09, 0.23) |
| B | -0.01 | (-0.35, 0.36) | 0.00 | (-0.05, 0.05) | 1.66 | (0.38, 2.90) | 0.22 | (0.04, 0.39) |
| All influenza | -0.13 | (-0.60, 0.46) | 0.03 | (-0.04, 0.11) | 2.53 | (0.79, 4.21) | 0.38 | (0.12, 0.61) |
|  |  |  |  |  |  |  |  |  |
| **Male + Female** |  |  |  |  |  |  |  |  |
| A(H1N1) | -0.04 | (-0.23, 0.18) | 0.00 | (-0.03, 0.04) | 0.07 | (-0.66, 0.81) | 0.04 | (-0.07, 0.14) |
| A(H3N2) | -0.04 | (-0.31, 0.26) | 0.01 | (-0.03, 0.06) | 0.39 | (-0.39, 1.31) | 0.02 | (-0.10, 0.15) |
| B | -0.09 | (-0.39, 0.21) | 0.04 | (-0.01, 0.09) | 1.06 | (0.03, 2.01) | 0.18 | (0.03, 0.31) |
| All influenza | -0.17 | (-0.61, 0.28) | 0.05 | (-0.03, 0.13) | 1.53 | (0.10, 2.86) | 0.23 | (0.04, 0.40) |

Table S3. Average type and subtype-specific annual excess septicemia hospitalization rates in different sex and age groups in Hong Kong, 1998 to 2019, assuming 0-week lag between influenza incidence and hospitalization.

|  | Average excess hospitalization rate (per 100,000 population per year) | | | | | | | |
| --- | --- | --- | --- | --- | --- | --- | --- | --- |
| Virus | 0-4 y | (95% CI) | 5-64 y | (95% CI) | ≥65 y | (95% CI) | All Ages | (95% CI) |
| **Male** |  |  |  |  |  |  |  |  |
| A(H1N1) | 4.39 | (-0.37, 9.26) | 0.07 | (-0.29, 0.43) | -0.69 | (-4.20, 2.80) | 0.11 | (-0.42, 0.67) |
| A(H3N2) | 1.32 | (-6.27, 6.70) | 0.32 | (-0.13, 0.76) | 6.35 | (2.63, 10.92) | 0.96 | (0.24, 1.64) |
| B | -1.12 | (-8.03, 4.71) | 0.33 | (-0.10, 0.79) | 1.14 | (-3.12, 5.92) | 0.31 | (-0.45, 1.09) |
| All influenza | 4.59 | (-6.28, 12.33) | 0.72 | (0.11, 1.30) | 6.79 | (0.91, 13.46) | 1.38 | (0.28, 2.37) |
|  |  |  |  |  |  |  |  |  |
| **Female** |  |  |  |  |  |  |  |  |
| A(H1N1) | 3.12 | (-1.26, 8.97) | -0.02 | (-0.24, 0.23) | -3.93 | (-7.27, -0.31) | -0.36 | (-0.93, 0.18) |
| A(H3N2) | 9.34 | (2.30, 14.55) | 0.24 | (-0.04, 0.51) | 5.88 | (2.19, 10.15) | 1.36 | (0.74, 2.03) |
| B | 4.94 | (-2.87, 11.16) | 0.08 | (-0.23, 0.40) | 5.61 | (1.44, 10.22) | 0.98 | (0.30, 1.74) |
| All influenza | 17.40 | (7.06, 25.41) | 0.30 | (-0.14, 0.72) | 7.55 | (2.14, 14.47) | 1.97 | (1.08, 3.02) |
|  |  |  |  |  |  |  |  |  |
| **Male + Female** |  |  |  |  |  |  |  |  |
| A(H1N1) | 3.70 | (0.03, 7.69) | 0.03 | (-0.17, 0.23) | -2.51 | (-5.20, 0.17) | -0.13 | (-0.55, 0.31) |
| A(H3N2) | 5.44 | (0.02, 10.07) | 0.28 | (0.01, 0.54) | 5.99 | (3.06, 9.58) | 1.19 | (0.65, 1.69) |
| B | 2.25 | (-3.25, 7.10) | 0.18 | (-0.07, 0.43) | 3.62 | (0.47, 7.30) | 0.68 | (0.16, 1.24) |
| All influenza | 11.39 | (3.87, 18.10) | 0.50 | (0.11, 0.84) | 7.09 | (2.85, 12.92) | 1.73 | (0.94, 2.50) |

Table S4. Average type and subtype-specific annual excess septicemia mortality rates in all-ages in Hong Kong, 1998 to 2019.
Alternative lags, ranging from 0 to 3 weeks, were assumed between influenza incidence and mortality.

|  | Average excess mortality rate (per 100,000 population per year) | | | | | | | | |
| --- | --- | --- | --- | --- | --- | --- | --- | --- | --- |
| Virus | 0-week lag | (95% CI) | 1-week lag | (95% CI) | 2-week lag | (95% CI) | 3-week lag | (95% CI) |  |
| **Male** |  |  |  |  |  |  |  |  |  |
| A(H1N1) | 0.04 | (-0.11, 0.15) | -0.04 | (-0.18, 0.08) | -0.08 | (-0.22, 0.03) | -0.07 | (-0.20, 0.07) |  |
| A(H3N2) | 0.06 | (-0.08, 0.25) | 0.02 | (-0.12, 0.21) | -0.03 | (-0.17, 0.17) | -0.02 | (-0.15, 0.18) |  |
| B | 0.19 | (0.03, 0.37) | 0.08 | (-0.08, 0.27) | 0.02 | (-0.13, 0.21) | 0.04 | (-0.14, 0.22) |  |
| All influenza | 0.29 | (0.06, 0.55) | 0.06 | (-0.16, 0.32) | -0.09 | (-0.30, 0.19) | -0.05 | (-0.28, 0.25) |  |
|  |  |  |  |  |  |  |  |  |  |
| **Female** |  |  |  |  |  |  |  |  |  |
| A(H1N1) | 0.13 | (-0.01, 0.27) | 0.09 | (-0.03, 0.21) | 0.05 | (-0.08, 0.18) | -0.02 | (-0.15, 0.12) |  |
| A(H3N2) | 0.06 | (-0.09, 0.23) | 0.07 | (-0.09, 0.23) | 0.10 | (-0.06, 0.26) | 0.11 | (-0.06, 0.27) |  |
| B | 0.32 | (0.13, 0.49) | 0.22 | (0.04, 0.39) | 0.10 | (-0.08, 0.27) | 0.09 | (-0.08, 0.27) |  |
| All influenza | 0.51 | (0.25, 0.76) | 0.38 | (0.12, 0.61) | 0.24 | (-0.03, 0.51) | 0.18 | (-0.08, 0.44) |  |
|  |  |  |  |  |  |  |  |  |  |
| **Male + Female** |  |  |  |  |  |  |  |  |  |
| A(H1N1) | 0.10 | (-0.02, 0.21) | 0.04 | (-0.07, 0.14) | -0.01 | (-0.11, 0.09) | -0.05 | (-0.15, 0.07) |  |
| A(H3N2) | 0.04 | (-0.08, 0.17) | 0.02 | (-0.10, 0.15) | 0.02 | (-0.09, 0.15) | 0.03 | (-0.09, 0.17) |  |
| B | 0.28 | (0.14, 0.42) | 0.18 | (0.03, 0.31) | 0.07 | (-0.06, 0.20) | 0.07 | (-0.07, 0.21) |  |
| All influenza | 0.42 | (0.23, 0.62) | 0.23 | (0.04, 0.40) | 0.08 | (-0.10, 0.28) | 0.06 | (-0.13, 0.27) |  |

Table S5. Average type and subtype-specific annual excess septicemia hospitalization rates in all-ages in Hong Kong, 1998 to 2019.
Alternative lags, ranging from 0 to 2 weeks, were assumed between influenza incidence and hospitalization.

|  | Average excess hospitalization rate (per 100,000 population per year) | | | | | | |
| --- | --- | --- | --- | --- | --- | --- | --- |
| Virus | 0-week lag | (95% CI) | 1-week lag | (95% CI) | 2-week lag | (95% CI) |  |
| **Male** |  |  |  |  |  |  |  |
| A(H1N1) | 0.11 | (-0.42, 0.67) | 0.00 | (-0.57, 0.56) | -0.03 | (-0.61, 0.56) |  |
| A(H3N2) | 0.96 | (0.24, 1.64) | 0.78 | (0.04, 1.46) | 0.61 | (-0.05, 1.27) |  |
| B | 0.31 | (-0.45, 1.09) | 0.11 | (-0.67, 0.83) | 0.35 | (-0.43, 1.13) |  |
| All influenza | 1.38 | (0.28, 2.37) | 0.89 | (-0.24, 1.90) | 0.94 | (-0.16, 1.96) |  |
|  |  |  |  |  |  |  |  |
| **Female** |  |  |  |  |  |  |  |
| A(H1N1) | -0.36 | (-0.93, 0.18) | -0.39 | (-0.93, 0.15) | -0.41 | (-0.94, 0.16) |  |
| A(H3N2) | 1.36 | (0.74, 2.03) | 1.42 | (0.83, 2.10) | 1.12 | (0.59, 1.82) |  |
| B | 0.98 | (0.30, 1.74) | 0.89 | (0.22, 1.75) | 0.94 | (0.28, 1.78) |  |
| All influenza | 1.97 | (1.08, 3.02) | 1.93 | (1.06, 3.06) | 1.65 | (0.84, 2.83) |  |
|  |  |  |  |  |  |  |  |
| **Male + Female** |  |  |  |  |  |  |  |
| A(H1N1) | -0.13 | (-0.55, 0.31) | -0.19 | (-0.60, 0.21) | -0.22 | (-0.66, 0.22) |  |
| A(H3N2) | 1.19 | (0.65, 1.69) | 1.16 | (0.65, 1.70) | 0.92 | (0.46, 1.41) |  |
| B | 0.68 | (0.16, 1.24) | 0.56 | (0.04, 1.17) | 0.70 | (0.14, 1.28) |  |
| All influenza | 1.73 | (0.94, 2.50) | 1.53 | (0.78, 2.35) | 1.41 | (0.68, 2.22) |  |

**3. Temperature and absolute humidity as confounders**

Temporal trends of temperature and absolute humidity are presented in Figure S3. Particularly, a clear and well-defined seasonal trend is observed for both temperature and absolute humidity, where peaks during the annual summer seasons were experienced before dropping to its troughs at the beginning of every year. Humidity and temperature were indicated to affect survival and transmissibility of influenza viruses in experimental studies, and associated with mortality and hospitalization in Hong Kong, and we included them as covariates in the regression models.

Figure S3. Seasonal trends of temperature and absolute humidity in Hong Kong,
1998-2019.
